# Role of Serine Racemase in Behavioral Sensitization in Mice after Repeated Administration of Methamphetamine

**DOI:** 10.1371/journal.pone.0035494

**Published:** 2012-04-18

**Authors:** Mao Horio, Mami Kohno, Yuko Fujita, Tamaki Ishima, Ran Inoue, Hisashi Mori, Kenji Hashimoto

**Affiliations:** 1 Division of Clinical Neuroscience, Chiba University Center for Forensic Mental Health, Chiba, Japan; 2 Department of Molecular Neuroscience, Toyama University Graduate School of Medicine, Toyama, Japan; Centre for Addiction and Mental Health, Canada

## Abstract

**Background:**

The *N*-methyl-D-aspartate (NMDA) receptors play a role in behavioral abnormalities observed after administration of the psychostimulant, methamphetamine (METH). Serine racemase (SRR) is an enzyme which synthesizes D-serine, an endogenous co-agonist of NMDA receptors. Using *Srr* knock-out (KO) mice, we investigated the role of SRR on METH-induced behavioral abnormalities in mice.

**Methodology/Principal Findings:**

Evaluations of behavior in acute hyperlocomotion, behavioral sensitization, and conditioned place preference (CPP) were performed. The role of SRR on the release of dopamine (DA) in the nucleus accumbens after administration of METH was examined using *in vivo* microdialysis technique. Additionally, phosphorylation levels of ERK1/2 proteins in the striatum, frontal cortex and hippocampus were examined using Western blot analysis. Acute hyperlocomotion after a single administration of METH (3 mg/kg) was comparable between wild-type (WT) and *Srr*-KO mice. However, repeated administration of METH (3 mg/kg/day, once daily for 5 days) resulted in behavioral sensitization in WT, but not *Srr*-KO mice. Pretreatment with D-serine (900 mg/kg, 30 min prior to each METH treatment) did not affect the development of behavioral sensitization after repeated METH administration. In the CPP paradigm, METH-induced rewarding effects were demonstrable in both WT and *Srr*-KO mice. *In vivo* microdialysis study showed that METH (1 mg/kg)-induced DA release in the nucleus accumbens of *Srr*-KO mice previously treated with METH was significantly lower than that of the WT mice previously treated with METH. Interestingly, a single administration of METH (3 mg/kg) significantly increased the phosphorylation status of ERK1/2 in the striatum of WT, but not *Srr*-KO mice.

**Conclusions/Significance:**

These findings suggest first, that SRR plays a role in the development of behavioral sensitization in mice after repeated administration of METH, and second that phosphorylation of ERK1/2 by METH may contribute to the development of this sensitization as seen in WT but not *Srr-*KO mice.

## Introduction

Abuse of the psychostimulant methamphetamine (METH) is a serious and growing worldwide problem. Long-term use of METH results in addiction, which is characterized by compulsive drug-seeking and drug use, with accompanying functional and molecular changes in the brain. Addiction to METH is also a major public health concern, since chronic use is associated with major medical, psychiatric, cognitive, socioeconomic and legal consequence [1], [2]. Repeated consumption of METH can induce a psychotic state (METH psychosis), with symptoms resembling those of paranoid-type schizophrenia [3]–[5]. There is currently no standard pharmacological treatment for the wide range of symptoms associated with METH abuse [1], [6], [7]. Moreover, the precise molecular and cellular mechanisms underlying the long-term effects of METH in the brain, remain undetermined [1], [8], [9].

METH causes neurochemical changes in several areas of the brain via the dopaminergic system, and consequently via glutamatergic neurotransmission [10]–[13]. Repeated administration of this psychostimulant to rodents, produces long-term behavioral changes, including behavioral sensitization and dependence [14], [15]. The *N*-methyl-D-aspartate (NMDA) receptor antagonist MK-801 blocks the development of METH (or amphetamine)-induced behavioral sensitization [16]–[20]. It is therefore likely that the NMDA receptor plays a role in the mechanisms of behavioral sensitization seen in rodents after repeated administration of psychostimulants, such as METH and amphetamine.

D-Serine is an endogenous co-agonist at the glycine-binding site of the NMDA receptor subunit, GluN1 [21]–[23]. D-Serine is synthesized from L-serine by the enzyme, serine racemase (SRR), and shows a similar localization within the brain to D-serine [24], [25]. Studies using *Srr* knockout (*Srr*-KO) mice show that SRR is predominantly localized to forebrain neurons [26] and that levels of D-serine in the forebrain are 10–20% of wild-type (WT) mice [27]–[29], suggesting that SSR provides the main catalysis for D-serine production in the forebrain. In addition, we reported that NMDA-induced neurotoxicity is significantly attenuated in the brains of *Srr*-KO mice, suggesting that D-serine controls the extent of NMDA receptor-mediated neurotoxic insults [27]. It is therefore likely that D-serine produced by SSR, plays an important role in NMDA receptor-mediated neurotransmission in the brain.

To study the role of SRR in METH-induced behavioral abnormalities, we evaluated behavioral performances in acute hyperlocomotion, development of behavioral sensitization, and conditioned place preference (CPP) in WT and *Srr*-KO mice, after the administration of METH. Furthermore, we examined the role of SRR on the dopamine (DA) release in the nucleus accumbens after administration of METH using *in vivo* microdialysis technique. In addition, we examined whether METH administration altered phosphorylation levels of ERK1/2 in the striatum, since ERK1/2 phosphorylation contributes to the development of behavioral sensitization by psychostimulants [30], [31].

## Results

### METH-induced acute hyperlocomotion

**Figure 1:**
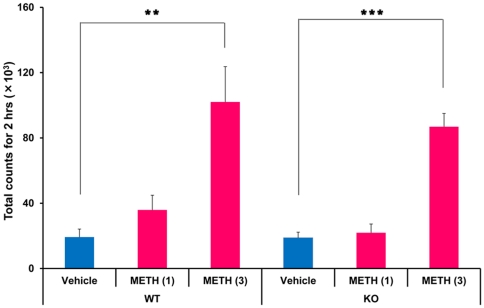
METH-induced acute hyperlocomotion in WT and Srr-KO mice. METH (1 or 3 mg/kg) or vehicle (saline; 10 ml/kg) was administered s.c. to WT and Srr-KO mice. Behavior (locomotion) was evaluated as described in the Methods and Materials. Each value is the mean ± SEM (n = 8–10 per group). **p<0.01, ***p<0.001 as compared with the vehicle treated group.

A single dose of METH (3 mg/kg, s.c.), but not METH (1 mg/kg, s.c.), markedly increased locomotion in both WT and *Srr*-KO mice. Two-way ANOVA analysis revealed significant drug treatment effects for METH-induced locomotor responses [genotype: F (1,48) = 1.12, p = 0.29; drug treatment: F (2,48) = 26.97, p<0.001], with no genotype x drug treatment interaction (F (2,48) = 0.27, p = 0.77). Subsequent one-way ANOVA followed *post hoc* Bonferroni/Dun test indicated that METH (3 mg/kg) significantly increased locomotion in both WT and *Srr-*KO mice (WT: p* = *0.002; *Srr*-KO: p<0.001 as compared to saline treated group) (**Figure 1**).

Next, we examined whether pretreatment with D-serine affected METH-induced acute hyperlocomotion in mice. Thirty minutes after a single oral dose of D-serine (900 mg/kg) or vehicle (10 ml/kg), mice were given a s.c dose of METH (3 mg/kg). Two-way ANOVA analysis revealed that D-serine had no significant effect on METH-induced acute hyperlocomotion [genotype: F (1,24) = 6.00, p = 0.02; drug treatment: F (1,24) = 0.02, p = 0.88; interaction: F(1,24) = 0.02, p = 0.89]. Student's t-test indicated that pretreatment with D-serine (900 mg/kg) had no effect on METH-induced hyperlocomotion in either WT or *Srr*-KO mice (WT: t = 0.26, p* = *0.80; *Srr*-KO: t = 0.006, p = 1.00) (**Figure S1**).

### METH-induced behavioral sensitization

**Figure 2:**
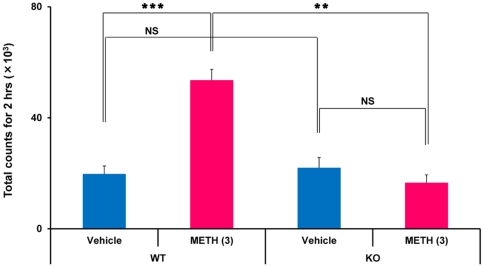
The development of behavioral sensitization in mice after repeated administration of METH. Mice were treated daily for 5 consecutive days with vehicle (10 ml/kg) or METH (3 mg/kg). Seven days after the final dosing, mice were given a lower dose of METH (1 mg/kg, s.c.). Locomotion in mice was evaluated as described in the Methods and Materials. Each value is the mean ± SEM (n = 9 or 10 per group). **p<0.01, ***p<0.001 as compared with the vehicle treated group (Bonferroni/Dunn method).

Two-way ANOVA analysis revealed a significant effect for METH-induced hyperlocomotion [genotype: F (1,34) = 7.97, p = 0.008; drug treatment: F (1,34) = 33.46, p<0.001; interaction: F (1,34) = 11.83, p = 0.002]. One-way ANOVA revealed a significant (F (3,34) = 18.37, p<0.001) difference among the four groups. Challenging mice with a low dose of METH (1 mg/kg) significantly (p<0.001) increased METH -induced hyperlocomotion in WT mice previously treated with METH (3 mg/kg/day over 5 consecutive days), compared with saline treated WT mice (**Figure 2**). In contrast, METH (1 mg/kg) -induced hyperlocomotion was comparable between *Srr*-KO mice previously treated with METH (3 mg/kg/day over 5 consecutive days) or saline (**Figure 2**), indicating a lack of METH-induced behavioral sensitization in *Srr*-KO mice. Furthermore, locomotion in WT mice pretreated with METH (3 mg/kg/day for 5 consecutive days) was significantly (p = 0.001) higher than seen in *Srr*-KO mice pretreated with METH (**Figure 2**). These results indicated that behavioral sensitization occurred in WT but not *Srr*-KO mice after repeated administration of METH.

To examine whether pretreatment with D-serine affected METH-induced behavioral sensitization in *Srr*-KO mice, mice were administered a single oral dose of D-serine (900 mg/kg/day) or vehicle (10 ml/kg/day) thirty minutes before dosing with METH (3 mg/kg/day). Two-way ANOVA analysis revealed a significant genotypic effect for METH-induced locomotion [genotype: F (1,24) = 27.17, p<0.001; drug treatment: F (1,24) = 1.31, p = 0.26], with no genotype x drug interaction (F (1,24) = 0.21, p = 0.65). One-way ANOVA revealed significant (F (3,24) = 9.562, p<0.001) differences among the four groups. Pretreatment with D-serine (900 mg/kg) showed no effect on METH (1 mg/kg)-induced locomotion in either WT or *Srr-*KO mice. In contrast, locomotion in WT mice pretreated with vehicle followed by METH (3 mg/kg) was significantly (p = 0.015) higher than in *Srr*-KO mice treated in the same way (**Figure S2**), consistent with results in **Figure 2**. Furthermore, locomotion in WT mice pretreated with D-serine (900 mg/kg) followed by a larger dose of METH (3 mg/kg) was significantly (p = 0.003) higher than in *Srr*-KO mice treated in the same manner (**Figure S2**). These results showed that pretreatment of D-serine (900 mg/kg) prior to each METH injection had no effect on METH (1 mg/kg)-induced locomotion in WT and *Srr*-KO mice pretreated with METH.

### METH-induced DA release in the nucleus accumbens

**Figure 3:**
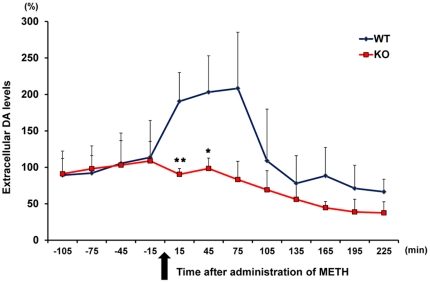
The effects of SRR on METH-induced increase of extracellular DA levels. A dose of METH (1.0 mg/kg, s.c) was injected into mice. The dialysate was collected in 30-min fractions, and DA levels were measured by HPLC. Basal extracellular DA levels in the nucleus accumbens were 3.841±0.301 nmol/L (n = 12, mean ± SEM). The values are the mean ± SEM of 6 mice. *p<0.05, **p<0.01 as compared with the METH (1 mg/kg) treated WT group (Student's t-test).

To explore how SRR contributes to METH-induced sensitization, we measured extracellular DA levels in the nucleus accumbens after administration of METH (1 mg/kg), using an *in vivo* microdialysis technique. A dose of METH (1 mg/kg, s.c.) caused a marked increase in extracellular DA levels in the nucleus accumbens of WT, not *Srr*-KO, mice previously treated with METH (3 mg/kg/day over 5 consecutive days) (**Figure 3**). Repeated ANOVA analysis showed a significant difference between two groups (Time×Group, F = 3.456, p = 0.042). The METH-induced DA release in the nucleus accumbes of *Srr*-KO mice previously treated with METH was significantly lower than that of WT mice previously treated with METH (**Figure 3**). These findings suggest that an administration of METH (1 mg/kg) failed to induce DA release in the nucleus accumbens of *Srr*-KO mice previously treated with METH.

### METH-induced rewarding effects

**Figure 4:**
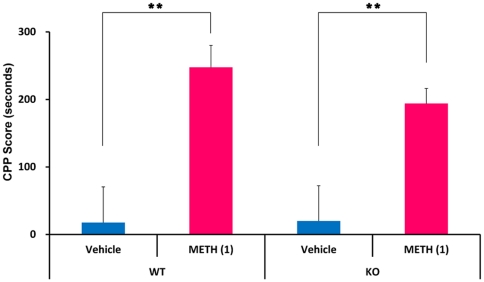
METH-induced conditioned place preference (CPP) in mice. On days 4, 6, and 8, mice were treated with vehicle (10 ml/kg) or METH (1 mg/kg), and then confined in either a transparent or black compartment for 30 min. On days 5, 7, and 9, mice were given saline and placed in the non- METH assigned compartment for 30 min. On day 10, the postconditioning test was performed as described in the Methods and Materials. Each value is the mean ± SEM (n = 11 per group). **p<0.01 as compared with the vehicle treated group (Student's t-test).

Mice that had only received METH spent significantly more time in the METH assigned compartment relative to the saline treatment compartment. The mice conditioned with saline did not show a preference for either compartment on the CPP test day. These data showed that a single pairing with METH (1 mg/kg, s.c.)-induced CPP when mice were tested 24 h after conditioning. Two-way ANOVA analysis revealed a significant drug treatment effect for the METH-induced CPP score [genotype: F (1,40) = 0.37, p = 0.55; drug treatment: F (1,40) = 23.11, p<0.001], with no genotype x drug treatment interaction (F(1,40) = 0.44, p = 0.51). Student's t-test indicated that repeated administration of METH (1 mg/kg) significantly increased CPP scores in both WT and *Srr*-KO mice (WT: t = 3.71, p = 0.001; *Srr*-KO: t = 3.07, p = 0.006) (**Figure 4**). The data indicated that METH treatment induced rewarding effects in both WT mice and *Srr*-KO mice.

### Phosphorylation of ERK1/2

**Figure 5:**
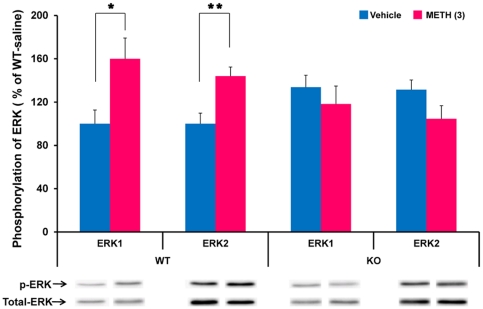
Phosphorylation of ERK1/2 in the striatum after a single dose of METH. Mice were sacrificed 15 minutes after a single dose of either METH (3 mg/kg, s.c.) or vehicle (10 ml/kg, s.c.). Western blot analysis of phospho-ERK1/2 and total ERK1/2 protein was performed as described in the Methods and Materials. Values are the mean ± S.E.M. (n = 6 per group). *p<0.05, **p<0.01 as compared with the vehicle treated group (Student's t-test).

ERK, a component of the mitogen-activated protein kinase (MAPK) intracellular signaling pathway, interacts with both dopamine and NMDA receptors in the brain. It has been reported that the phosphorylation of ERK1/2 plays a role in behavioral sensitization, after repeated administration of psychostimulants [30], [31]. This study examined whether phosphorylation levels of ERK1/2 in the striatum of *Srr*-KO mice differed from that of WT mice. Two-way ANOVA analysis revealed a significant effect for METH-induced phosphorylation of ERK1/2 between WT and *Srr-*KO mice [ERK1; genotype: F (1,20) = 0.07, p = 0.80; drug treatment: F (1,20) = 2.12, p = 0.16; interaction: F (1,20) = 6.13, p = 0.02; ERK2; genotype: F (1,20) = 0.16, p = 0.70; drug treatment: F (1,20) = 0.75, p = 0.40; interaction: F (1,20) = 12.89, p = 0.002]. Student's t-test indicated that a single dose of METH (3 mg/kg) significantly increased the phosphorylation of ERK1/2 in the WT mice [ERK1; WT: t = 2.61, p* = *0.03; ERK2; WT: t = 3.41, p* = *0.006] (**Figure 5**). In contrast, a single dose of METH (3 mg/kg) did not increase phosphorylation of ERK1/2 in *Srr-*KO mice (ERK1; *Srr*-KO: t = 0.78, p = 0.46; ERK2; *Srr*-KO: t = 1.79, p = 0.10) (**Figure 5**).

In the hippocampus, two-way ANOVA analysis revealed no significant effect for METH-induced phosphorylation of ERK1/2 between WT and *Srr*-KO mice [ERK1; genotype: F (1,20) = 0.42, p = 0.53; drug treatment: F (1,20) = 0.23, p = 0.64; interaction: F (1,20) = 2.54, p = 0.13; ERK2; genotype: F (1,20) = 7.23, p = 0.01; drug treatment: F (1,20) = 1.61, p = 0.21; interaction: F (1,20) = 0.41, p = 0.53](**Figure S3**). In the frontal cortex, the phosphorylation of ERK1/2 was not detected between WT and *Srr* ***-***KO mice (data not shown).

## Discussion

The major findings of this study are that repeated administration of METH (3 mg/kg/day for 5 days) induced behavioral sensitization in WT mice, but not *Srr*-KO mice, and that a single dose of METH (3 mg/kg) produced the same changes in acute hyperlocomotion between *Srr*-KO and WT mice. From *in vivo* microdialysis, we found that METH-induced DA release in the nucleus accumbens of *Srr*-KO mice previously treated with METH (3 mg/kg/day over 5 consecutive days) was significantly attenuated as compared with WT mice previously treated with METH (3 mg/kg/day over 5 consecutive days). To our knowledge, this is the first report demonstrating the role of SRR in the development of METH-induced behavioral sensitization. Previously, we reported that levels of D-serine in the forebrain of *Srr*-KO mice were reduced to approximately 10–20% of those found in WT mice [27], [29]. However, pretreatment with D-serine (900 mg/kg) did not alter hyperlocomotion in WT and *Srr*-KO mice after a single dose of METH (3 mg/kg), suggesting that decreased levels of D-serine in the brain do not affect METH-induced, acute hyperlocomotion in mice. Furthermore, pretreatment with D-serine (900 mg/kg/day) prior to each METH injection, failed to induce behavioral sensitization in *Srr*-KO mice. It is therefore unlikely that pretreatment with D-serine affects METH-induced behavioral abnormalities in either WT or *Srr*-KO mice. These findings suggest that SRR plays an important role in the development of behavioral sensitization in mice, following repeated METH administration.

Behavioral sensitization following the repeated administration of psychostimulants, including METH, is one manifestation of sensitization in the brain. The initiation of behavioral sensitization to psychostimulants is operationally defined as the transient sequence of cellular and molecular events precipitated by repeated administration of psychostimulants that leads to the enduring changes in neural function responsible for behavioral augmentation [14], [15], [32], [33]. The increase in extracellular DA is augmented in the nucleus accumbens and striatum following the repeated administration of psychostimulants. Together, the enhanced release of DA in the nucleus accumbens plays a role in the augmentation of behavior in the sensitized animals [14], [15], [32], [33]. In this study, we found that *Srr*-KO mice pretreated with METH did not show the release of DA in the nucleus accumbens and behavioral augmentation after a challenge of METH, indicating a role of *Srr* in the development of behavioral sensitization after repeated administration of METH.

Accumulating evidence suggests that intracellular signaling pathways, including that of ERK1/2 play contributes greatly to the molecular pathophysiology of drug addiction [34]–[38]. Abused drugs including amphetamine and cocaine has been shown to activate ERK in a subset of medium-sized spiny neurons of the dorsal striatum and nucleus accumbens, through the combined action of NMDA and DA D_1_ receptors [39]–[41]. Pretreatment with SL327 (30 mg/kg), a selective brain-penetrating inhibitor of MAP-kinase/ERK kinase, blocks the development of behavioral sensitization after repeated amphetamine treatment [39], suggesting a role for this pathway in long-lasting behavioral sensitization by psychostimulants. It has also been shown that phosphorylation of ERK1/2 in the striatum increases after administration of psychostimulants [30], [31], [40]–[41]. In this study, a single dose of METH (3 mg/kg) significantly increased the phosphorylation of ERK1/2 in the striatum of WT, but not *Srr*-KO mice. This suggests that phosphorylation of ERK1/2 in the striatum following a single dose of METH, is at least in part, mediated by SRR, although the precise mechanisms are currently unclear. Previously, we reported that NMDA-induced neurotoxicity is significantly attenuated in the brains of *Srr*-KO mice, suggesting that D-serine controls the extent of NMDA receptor-mediated neurotoxic insults [27]. It is likely that the lack of behavioral sensitization seen in *Srr*-KO mice may be the result of decreased DA release and ERK1/2 phosphorylation, due to NMDA receptor hypofunction, although this will need to be investigated further.

The CPP paradigm is a widely used animal model on the rewarding effects of drugs [42]. The NMDA receptor antagonist MK-801 fails to block amphetamine-induced place preference in rats, suggesting that NMDA receptors may not be involved in the rewarding effects of psychostimulants [43]. Miyamoto et al. [44] reported that mice with mutant GluN2A, one of four GluN2 subunits (GluN2A-D) of the NMDA receptor, developed METH-induced place preference to the same degree as WT mice, whereas behavioral sensitization was significantly reduced in these mutants compared with WT mice. This suggests that the GluN2A subunit may play a role in the development of behavioral sensitization, but not rewarding effects, in mice repeated exposure to METH. In this study, *Srr*-KO mice developed METH-induced place preference to the same degree as WT mice. It is therefore unlikely that NMDA receptors play a major role in the development of METH-induced rewarding effects in mice.

Along with D-serine, glycine is also a co-agonist at the glycine modulatory site of the NMDA receptor [21]. We reported that brain derived levels of glycine and other amino acids, including, glutamate and glutamine, were comparable between WT and *Srr*-KO mice [27], [29], suggesting that glycine may not compensate for decreased D-serine levels in the brains of *Srr*-KO mice. In addition, forebrain levels of the NMDA subunits, GluN2A, GluN2B, and GluN1 were comparable between WT and *Srr*-KO mice [27]. No difference was found in [^3^H](+)-MK-801 binding between brain regions (frontal cortex, hippocampus, striatum, cerebellum) from WT and *Srr*-KO mice (**Table S1**). It would seem that the expression of NMDA receptors is comparable between WT and *Srr*-KO mice. It is highly probable that the reduced D-serine in the forebrain of *Srr*-KO mice may, in part, contribute to the lack of METH-induced behavioral sensitization, although this too needs to be examined in further studies.

In conclusion, this study has pointed to a role for SRR in the development of behavioral sensitization, but not rewarding effects, in mice that have been repeatedly exposed to METH. It also suggests that decreased DA release in the nucleus accumbens and decreased phosphorylation of the ERK1/2 protein may contribute to the lack of METH-induced behavioral sensitization in *Srr*-KO mice.

## Materials and Methods

### Animals

*Srr*-KO mice were generated from C57BL/6- derived embryonic stem cells transfected with a gene-targeting vector containing C57BL/6 mouse genomic DNA, and the colony was expanded by crossing with C57BL/6 mice [26]. The generation and genotyping of *Srr*-KO and WT mice with a pure C57BL/6 genetic background has been reported previously [26]. WT and *Srr*-KO mice aged 2–3 months were used for all behavioral studies. The mice were housed in clear polycarbonate cages (22.5×33.8×14.0 cm) in groups of 5 or 6 per cage, under a controlled 12/12-h light–dark cycle (lights on from 7:00 AM to 7:00 PM), with a room temperature of 23±1°C and humidity at 55±5%. The mice were given free access to water and food pellets. The experimental procedure (Permit Number: #23-151) was approved by the Animal Care and Use Committee of Chiba University.

### Drug Administration

METH (D-methamphetamine hydrochloride) was purchased from Dainippon-Sumitomo Pharmaceutical Ltd., (Osaka, Japan) and D-serine from Sigma-Aldrich Corporation (St. Louis, MO). METH was dissolved in physiological saline and injected subcutaneously (s.c.). The dose of METH was expressed as a hydrochloride salt. D-Serine dissolved in saline was administered orally at a concentration of 900 mg/kg of body weight. Other chemicals were purchased from commercial sources.

### METH-induced Acute Hyperlocomotion

METH (1 and 3 mg/kg) or a vehicle of physiological saline (10 ml/kg) was administered s.c. into mice. Locomotor activity was measured using an animal movement analysis system (SCANET SV-10, Melquest, Toyama, Japan), as reported previously [45]–[47]. The system consisted of a rectangular enclosure (480×300 mm). The side walls (height, 60 mm) of the enclosure were equipped with 144 pairs of photosensors located 30 mm from the bottom edge and at 5 mm intervals. Recordings were taken from single animals. A pair of photosensors was scanned every 0.1 s to detect the animal movement. The intersection of paired photosensors (10 mm apart) in the enclosure was counted as one unit of locomotor activity. Data was collected for 180 min (60 min of habituation and for 120 min after the injection of METH or saline). To examine the role of D-serine in METH-induced acute hyperlocomotion, animals were pretreated with D-serine, before administration of METH. Thirty minutes after a single oral dose of D-serine (900 mg/kg) or vehicle (10 ml/kg), mice were administered a dose of METH (3 mg/kg, s.c). Locomotor activity was measured using the animal movement analysis system (SCANET SV-10, Melquest, Toyama, Japan), described above.

### METH-induced Behavioral Sensitization

Wild type and *Srr-*KO mice were given a single s.c. dose of vehicle (10 ml/kg) or METH (3 mg/kg), and returned to their home cages. This process was repeated for each animal, over 5 consecutive days. One week after the final treatment, each mouse was given a low dose of METH (1 mg/kg, s.c.), and behavioral changes (locomotion) were measured using the animal movement analysis system (SCANET SV-10, Melquest, Toyama, Japan), described above.

Next, mice were pretreated with D-serine in order to examine its role in METH-induced behavioral sensitization. Both WT and *Srr*-KO mice were treated with either: vehicle (10 ml/kg) + METH (3 mg/kg) group or D-serine (900 mg/kg) + METH (3 mg/kg) group. Injections were given 30 min apart. After the administration of METH, mice were returned to their home cages and this treatment was repeated over 5 consecutive days. One week after the final treatment, all mice were given a low dose of METH (1 mg/kg, s.c.), and behavioral changes (locomotion) were measured using the animal movement analysis system (SCANET SV-10, Melquest, Toyama, Japan), described above.

### Measurement of extracellular DA levels using *in vivo* microdialysis

Mice were anesthetized with sodium pentobarbital prior to the stereotaxic implantation of a probe into the nucleus accumbens (+1.1 mm anteroposterior, +1.0 mm mediolateral from the bregma, and −4.0 mm dorsoventral from the dura), according to the Franklin and Paxinos Atlas [48], as reported previously [49]. Probes were secured onto the skull using stainless-steel screws and dental acrylic. Twenty-four hours after surgery, *in vivo* microdialysis was performed on conscious and free moving mice. Probes were perfused continuously with artificial CSF (147 mM NaCl, 4 mM KCl, and 2.3 mM CaCl_2_) at a rate of 2 µl/min. METH (1 mg/kg, s.c.) was administered into mice. The dialysate was collected in 30-min fractions. After *in vivo* microdialysis experiments, the position of probe in the nucleus accumbens was confirmed in all mice. The DA levels in each fraction were measured by high performance liquid chromatography (HPLC), with electrochemical detection using a reversed phase column (EICOMPAK PP-ODS, 4.6×30 mm, Eicom, Kyoto, Japan) and a mobile phase 1% MeOH/100 mM phosphate buffer (pH 6.0) including 50 mg/L disodium EDTA disodium and 500 mg/L sodium decane-1-sulfonate.

### Conditioned Place Preference (CPP)

The place conditioning paradigm (CPP; Brain Science Idea Inc., Osaka, Japan) was used to study METH-induced rewarding effects, as reported previously [49]. Mice were allowed to move freely between transparent and black compartments for 15 min, once a day, for 3 days (days 1–3), as preconditioning. On day 3, the time spent in each compartment was measured. There was no significant difference between time spent in the black compartment with a smooth floor and the time spent in the transparent compartment with a textured floor, indicating that mice had no compartment preference before conditioning. On days 4, 6, and 8, mice were administered either vehicle (10 ml/kg, s.c.) or METH (1.0 mg/kg, s.c.), and then confined in either the transparent or black compartment for 30 min. On days 5, 7, and 9, the mice were given vehicle and placed in the non- METH assigned compartment, for 30 min. On day 10, the post-conditioning test was performed without drug treatment, and the time individual mice spent in each compartment was measured for 15 min. A counterbalanced protocol was used in order to nullify the initial preference of each mouse. The CPP score was designated as the time spent in the drug-conditioning sites minus the time spent in the saline-conditioning sites.

### Western Blot Analysis

In a preliminary experiment, we examined the time course for phosphorylation of ERK1/2 in the striatum, after a single dose of METH (3 mg/kg). We found increased phosphorylation of ERK1/2 15 min after a single METH administration (data not shown), consistent with a previous result [50]. In further experiments, mice were therefore sacrificed 15 min after dosing with either saline (10 ml/kg) or METH (3 mg/kg), then, the striatum, frontal cortex and hippocampus were dissected out on ice. Briefly, striatum from individual mice were frozen and homogenized in 500 µl of lysis buffer (20 mM TBS, pH 7.6, 10 mM NaF, 1 mM Na_3_VO_4_, 1% Triton x-100, 5 mM EDTA, 5 mM EGTA containing protease inhibitor) using a Polytron homogenizer. The sample was left to stand on ice for 30 min and then centrifuged at 10,000× g and 4°C for 30 min. Total protein in the supernatant was measured using the DC protein assay (Bio- Rad, Hercules, CA). The sample was then diluted with 5× SDS sample buffer (62.5 mM Tris-HCl, pH 6.8, 10% glycerol, 2% SDS, 5% β-mercaptoethanol and bromophenol blue). Aliquots (10 µg protein) of protein were incubated for 5 min at 95°C, then separated using SDS-PAGE on 12% polyacrylamide gels. Proteins were transferred for 1 h onto a polyvinylidene difluoride (PVDF) membrane (GE Healthcare Amersham Hybond™-P, UK), using Trans Blot Mini Cell apparatus (Bio-Rad, Hercules, CA). The transfer buffer consisted of 25 mM Tris and 192 mM glycine. After protein transfer, membranes were blocked for 45 min in TBS-T (20 mM Tris-HCl, pH 7.6, 137 mM NaCl, 0.1% Tween-20) containing 5% skimmed milk at RT, followed by incubation with anti-rabbit P44/42-ERK antibody (1∶1000, Cell Signaling, Cambridge, MA), overnight at 4°C in TBS-T, containing 5% BSA. After three washes in TBS-T, membranes were incubated with secondary antibody (1∶15,000) in TBS-T for 1 h at RT. After repeated washes, protein bands were detected using the ECL chemiluminescence detection system (GE Healthcare Bioscience, UK). Images were captured using a Fuji LAS3000-mini imaging system (Fujifilm, Tokyo, Japan), and chemiluminescence bands were quantified. To calculate the amount of phosphorylated protein relative to total protein, membranes were stripped in buffer (100 mM 2-mercaptoethanol, 2% SDS, and 62.5 mM Tris-HCl, pH 6.7) at 60°C for 30 min, washed, blocked, re-incubated with rabbit anti-ERK (1∶1000, Cell signaling, Cambridge, MA), and detected as described above.

### Statistical Analysis

All data were expressed as a mean ± standard error of the mean (S.E.M.). The behavior data, CPP score and ERK expression data were analyzed by two-way ANOVA (genotype vs. drug treatment). Student's t-test and one-way analysis of variance (ANOVA), followed Bonferroni/Dunn test were used for comparison between the two groups and comparison of multiple groups, respectively. The results of extracellular DA levels were analyzed by repeated one-way ANOVA, followed by the student's *t*-test. Values of *p*<0.05 were regarded as statistically significant.

## Supporting Information

Figure S1 **Effect of pretreatment with D-serine on acute hyperlocomotion after a single dose of METH.** Thirty minutes after a single oral dose of vehicle (10 ml/kg) or D-serine (900 mg/kg), WT and *Srr*-KO mice were given a dose of METH (3 mg/kg, s.c.). Behavioral evaluation of locomotion was performed 2 hours after the dose of METH, as described in the Methods and Materials section. Each value is the mean ± SEM (n = 7 per group). NS: Not significant (Student's t-test).
(TIFF)

Figure S2 **Effects of pretreatment with D-serine on behavioral sensitization after repeated administration of METH.** Thirty minutes after a single oral administration of vehicle (10 ml/kg) or D-serine (900 mg/kg), WT and *Srr*-KO mice were dosed with METH (3 mg/kg) for 5 consecutive days. Seven days after the final dose of METH, a lower dose of METH (1 mg/kg, s.c.) was administered to all mice. Behavioral evaluation of locomotion was performed. Each value is the mean ± SEM (n = 7 per group). ***p<0.01 as compared with the vehicle treated group (Bonferroni/Dunn method). NS: Not significant (Student's t-test).
(TIFF)

Figure S3 **Phosphorylation of ERK1/2 in the hippocampus after a single dose of METH.** Mice were sacrificed 15 minutes after a single dose of either METH (3 mg/kg, s.c.) or vehicle (10 ml/kg, s.c.). Western blot analysis of phospho-ERK1/2 and total ERK1/2 protein was performed as described in the Methods and Materials. Values are the mean ± S.E.M. (n = 6 per group).
(TIFF)

Table S1 **[^3^H](+)-MK-801 binding to mouse brain regions.** Binding of [^3^H](+)-MK-801 (3 nM; 1.02 TBq/mmol, PerkinElmer, MA, USA) to the crude membranes from brain regions (frontal cortex, hippocampus, striatum, cerebellum) was performed. Non-specific binding was determined in the presence of 10 µM of (+)-MK-801. There were no differences between WT mice and *Srr*-KO mice. Values are the mean ± S.E.M. (n = 7 per group).
(DOCX)
