# Supplementary figures and images for: Role of Serine Racemase in Behavioral Sensitization in Mice after Repeated Administration of Methamphetamine
Source: PLoS One. 2012 Apr 18;7(4):e35494. doi: 10.1371/journal.pone.0035494 (PMC3329469; doi:10.1371/journal.pone.0035494)

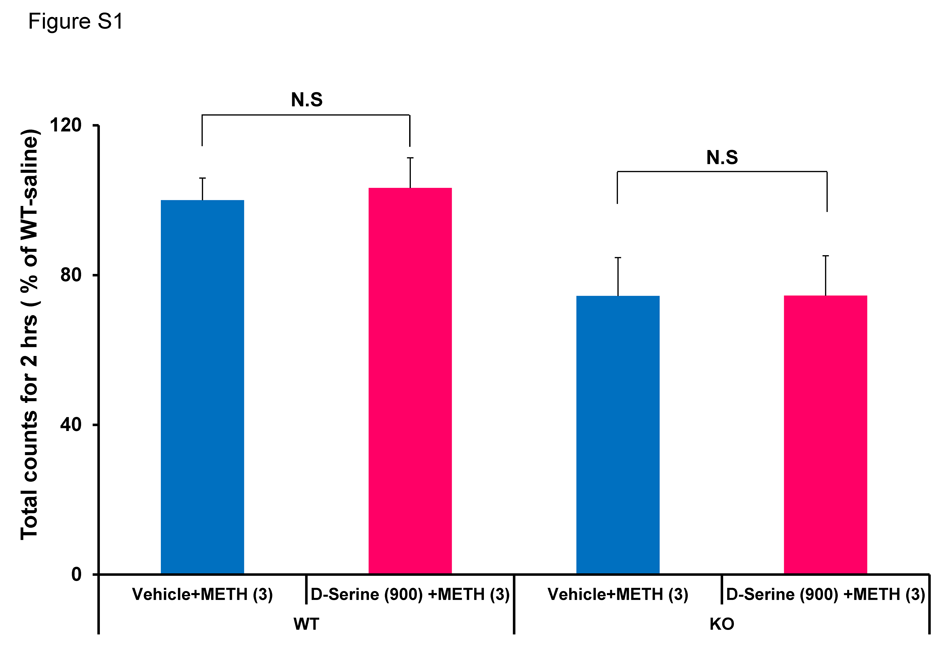

Supplement: Figure S1 — Effect of pretreatment with D-serine on acute hyperlocomotion after a single dose of METH. Thirty minutes after a single oral dose of vehicle (10 ml/kg) or D-serine (900 mg/kg), WT and Srr-KO mice were given a dose of METH (3 mg/kg, s.c.). Behavioral evaluation of locomotion was performed 2 hours after the dose of METH, as described in the Methods and Materials section. Each value is the mean ± SEM (n = 7 per group). NS: Not significant (Student's t-test). (TIFF) [file pone.0035494.s001.tiff]

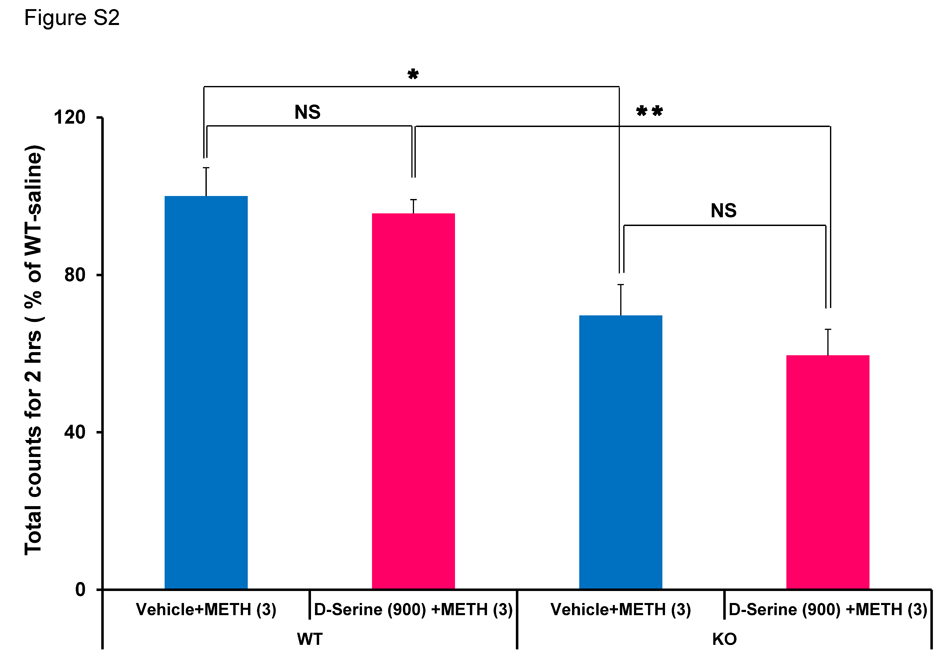

Supplement: Figure S2 — Effects of pretreatment with D-serine on behavioral sensitization after repeated administration of METH. Thirty minutes after a single oral administration of vehicle (10 ml/kg) or D-serine (900 mg/kg), WT and Srr-KO mice were dosed with METH (3 mg/kg) for 5 consecutive days. Seven days after the final dose of METH, a lower dose of METH (1 mg/kg, s.c.) was administered to all mice. Behavioral evaluation of locomotion was performed. Each value is the mean ± SEM (n = 7 per group). ***p<0.01 as compared with the vehicle treated group (Bonferroni/Dunn method). NS: Not significant (Student's t-test). (TIFF) [file pone.0035494.s002.tiff]

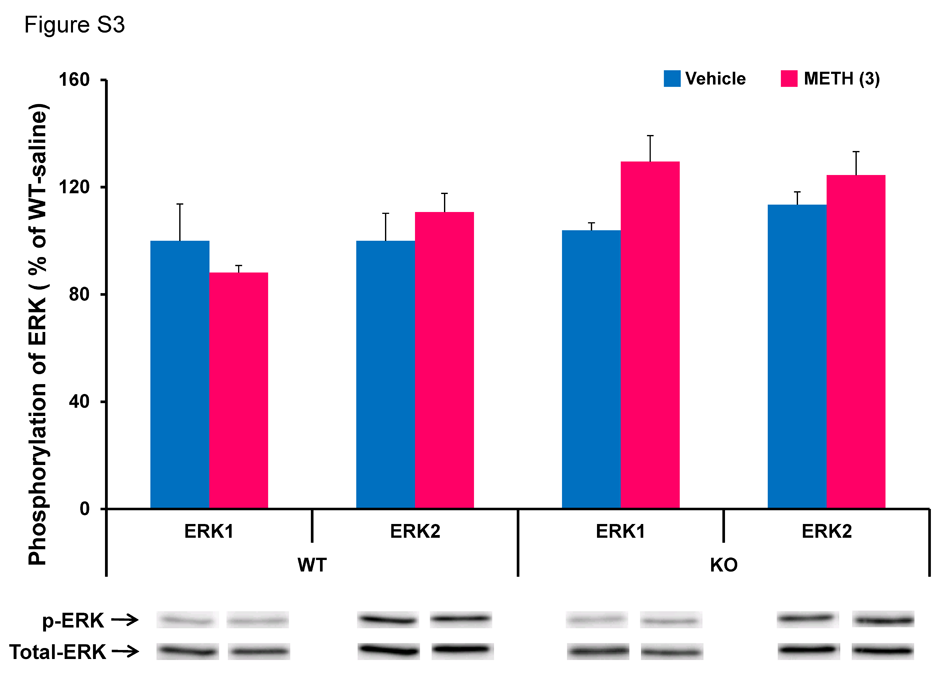

Supplement: Figure S3 — Phosphorylation of ERK1/2 in the hippocampus after a single dose of METH. Mice were sacrificed 15 minutes after a single dose of either METH (3 mg/kg, s.c.) or vehicle (10 ml/kg, s.c.). Western blot analysis of phospho-ERK1/2 and total ERK1/2 protein was performed as described in the Methods and Materials. Values are the mean ± S.E.M. (n = 6 per group). (TIFF) [file pone.0035494.s003.tiff]
