# Supplementary material for: Role of Serine Racemase in Behavioral Sensitization in Mice after Repeated Administration of Methamphetamine
Source: PLoS One. 2012 Apr 18;7(4):e35494. doi: 10.1371/journal.pone.0035494 (PMC3329469; doi:10.1371/journal.pone.0035494)
Supplement: Table S1 — [3H](+)-MK-801 binding to mouse brain regions. Binding of [3H](+)-MK-801 (3 nM; 1.02 TBq/mmol, PerkinElmer, MA, USA) to the crude membranes from brain regions (frontal cortex, hippocampus, striatum, cerebellum) was performed. Non-specific binding was determined in the presence of 10 µM of (+)-MK-801. There were no differences between WT mice and Srr-KO mice. Values are the mean ± S.E.M. (n = 7 per group). (DOCX) [file pone.0035494.s004.docx]

**Table S1. [^3^H](+)MK-801 binding to mouse brain regions**

|  |  | Specific binding |
| --- | --- | --- |
| Brain region | Genotype | (fmol/mg protein) |
| Frontal cortex | WT | 2.92 ± 0.08 |
|  | *Srr*-KO | 2.70 ± 0.03 |
| Hippocampus | WT | 2.68 ± 0.09 |
|  | *Srr*-KO | 2.69 ± 0.08 |
| Striatum | WT | 1.23 ± 0.05 |
|  | *Srr*-KO | 1.26 ± 0.04 |
| Cerebellum | WT | 0.19 ± 0.02 |
|  | *Srr*-KO | 0.15 ± 0.01 |

The values are the mean ± S.E.M. (n=7).

Specific binding of [^3^H](+)-MK-801 (3 nM; 1.02 TBq/mmol, PerkinElmer, MA, USA) to the brain membranes was determined in the presence of 10 μM of (+)-MK-801.
